# Unstably controlled systolic blood pressure trajectories are associated with markers for kidney damage in prediabetic population: results from the INDEED cohort study

**DOI:** 10.1186/s12967-020-02361-5

**Published:** 2020-05-12

**Authors:** Zi-Jun Sun, Jin-Wei Wang, Dong-Yuan Chang, Shuo-Hua Chen, Hui-Fen Zhang, Shou-Ling Wu, Kevin He, Lu-Xia Zhang, Min Chen, Ming-Hui Zhao

**Affiliations:** 1 Renal Division, Department of Medicine, Peking University First Hospital, Peking University Institute of Nephrology, Key Laboratory of Renal Disease, Ministry of Health of China, Key Laboratory of Chronic Kidney Disease Prevention and Treatment (Peking University), Ministry of Education, No. 8, Xishiku Street, Xicheng District, Beijing, 100034 China; 2 Health Care Center of Kailuan Group, Tangshan, 063000 China; 3 Laboratory Department of Kailuan General Hospital, Tangshan, 063000 China; 4 Department of Cardiology, Kailuan General Hospital Affiliated to North China University of Science and Technology, Tangshan, 063000 China; 5 Department of Biostatistics, School of Public Health, University of Michigan, Ann Arbor, MI USA; 6 Peking-Tsinghua Center for Life Sciences, Beijing, 100034 China

**Keywords:** Blood pressure, Estimated glomerular filtration rate, Kidney damage, Prediabetes, Trajectory

## Abstract

**Background:**

The association between blood pressure change and kidney damage in patients with abnormal blood glucose remains unclear. The current study aimed to identify systolic blood pressure (SBP) trajectories among the prediabetic population and to determine their association with kidney damage after a long-term follow-up.

**Methods:**

The incidence, development, and prognosis of diabetic kidney disease (INDEED) study is nested in the Kailuan cohort study with a focus on population with diabetes and prediabetes. We screened out people with prediabetes in 2006 and with more than three SBP records from 2006 to 2014 biennially. We used the latent mixture modeling to fit five groups of trajectories of SBP. In 2016, estimated glomerular filtration rate (eGFR), urinary albumin creatinine ratio (uACR), and urinary α1-microglobulin (α1MG), transferrin and α1-acid glycoprotein were measured, and the association between SBP trajectories and these markers was analyzed by linear regression and logistic regression models.

**Results:**

Totally, 1451 participants with prediabetes and without kidney damage were identified in 2006. Five heterogeneous SBP trajectories were detected based on the longitudinal data from 2006 to 2014, as low-stable group (n = 323), moderate-stable group (n = 726), moderate-increasing group (n = 176), moderate-decreasing group (n = 181), and high-stable group (n = 45). Linear regression analysis showed that the moderate and high SBP groups had lower eGFR, higher uACR, higher urinary α1MG, higher transferrin, and higher α1-acid glycoprotein than the low-stable group. Multivariable analysis attenuated the association but did not change the statistical significance.

**Conclusions:**

Prediabetic patients with persistent high-level SBP trajectory or gradually increased SBP trajectory had severer kidney damage during follow-up.

## Background

China is one of the countries with the highest prevalence of diabetes and prediabetes, which exert a heavy burden on social economy and health care. It was reported that the prevalence of prediabetes was 38.0% and 37.5% among adults in the United States and China, respectively [1, 2]. Prediabetes is a significant risk factor to develop hypertension, overt diabetes and cardiovascular disease (CVD) [3–8]. Previous studies found that the combination of prediabetes and hypertension is associated with a higher incidence of diabetes, CVD and chronic kidney disease (CKD) [5, 9], while the regression from prediabetes to normal glucose regulation can significantly reduce the CVD risk [10]. Besides, prediabetes might have an early renal lesion, such as the thickening of glomerular basement membrane [11], and it was an independent risk factor for glomerular hyperfiltration and could increase urinary albumin creatinine ratio (uACR), contributing to a poor kidney prognosis [8, 12, 13].

The optimal blood pressure (BP) target for patients with diabetes or prediabetes is not well defined [14, 15], and the relationship between hypertension and progression of prediabetes was unclear. Thus, to explore the influence of the longitudinal pattern of systolic blood pressure (SBP) in a population with prediabetes on the early stage of kidney damage and/or reduced kidney function is of great significance. The correlation between blood pressure merely at the baseline and the kidney disease may not adequately reflect their general and persistent association. The heterogeneity in effects may be observed for patterns of long-term blood pressure changes on the development and progression of diabetes and prediabetes. Based on the same cohort as the current study, Li et al. [16] analyzed the impact of SBP trajectories on qualitative measurements of urinary protein and reduced eGFR among diabetic patients. In the current study, we aimed to extend the research among the population with prediabetes and include the quantitatively measured spectrum of indicators, including estimated glomerular filtration rate (eGFR), uACR, and urinary α1-microglobulin (α1MG), transferrin and α1-acid glycoprotein, for early kidney injury and kidney function [17–20] after 10 years of follow-up.

## Methods

### Study design and participants

**Fig. 1:**
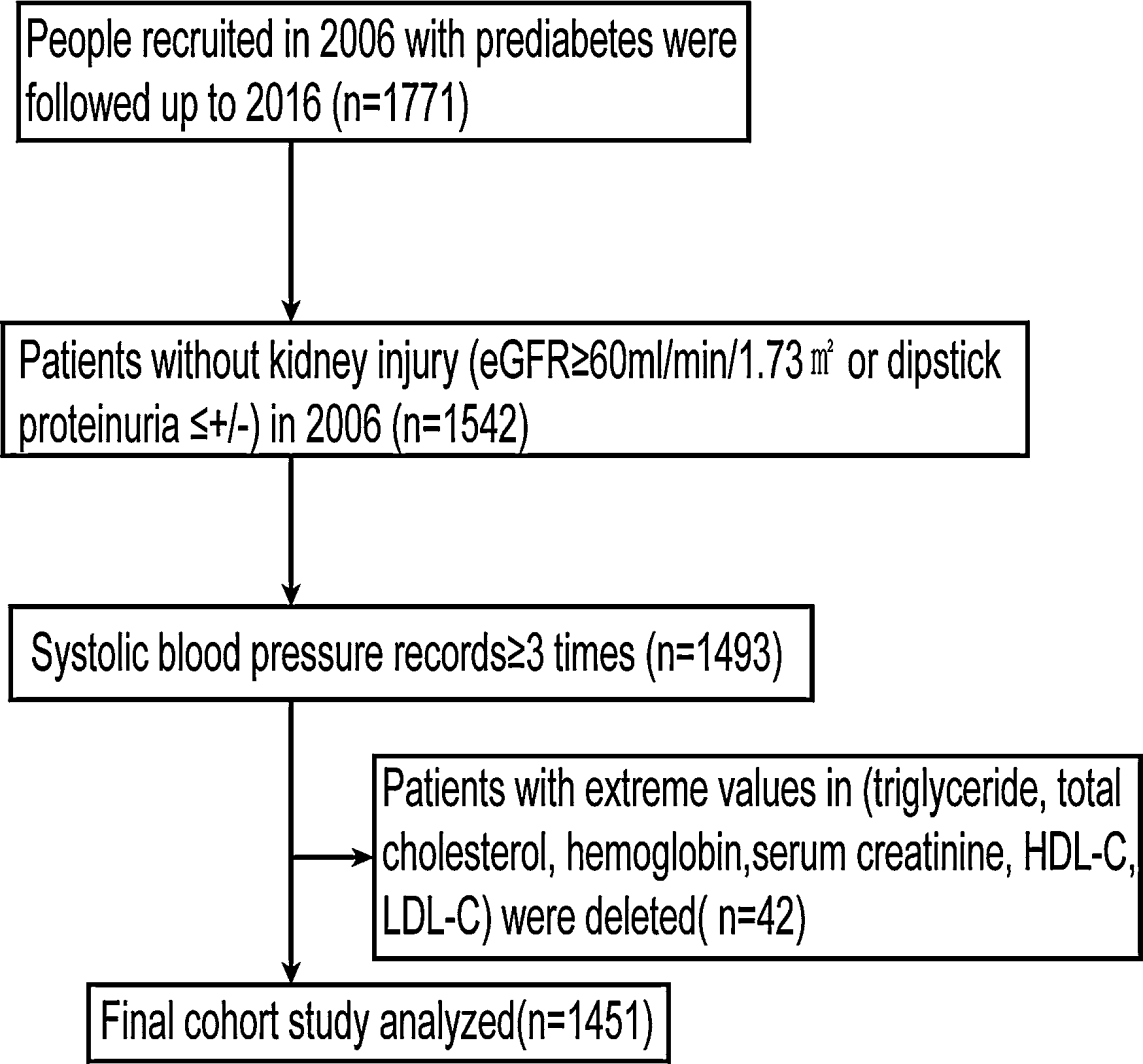
Flowchart of participants

The incidence, development, and prognosis of diabetic kidney disease (INDEED) study is based on the participants with diabetes and prediabetes in the Kailuan study, as described previously [21]. The Kailuan study, including 101,510 participants, is an ongoing prospective cohort study, which collected health records and related questionnaires from 11 hospitals in the Kailuan Community of Tangshan, Hebei Province of China. Briefly, all participants were followed up biennially from 2006 to update information according to the standard protocol [22]. In the current study, 1771 people were identified in 2006 with prediabetes and were followed-up until 2016. Prediabetes was defined as impaired fasting glucose (IFG) (≥ 5.6 mmol/L and ≤ 6.9 mmol/L) [23]. Participants with self-reported diabetes or self-reported current use of oral hypoglycemic medication or insulin were excluded. We excluded 229 people with kidney injury in 2006, defined as eGFR < 60 ml/min/1.73 m^2^ or urinary protein > ± with proteinuria dipstick. The above people with more than three times of systolic blood pressure records were retained. Forty-two participants who had missing value on total cholesterol (TCHO), triglyceride, high-density lipoprotein (HDL), low-density lipoprotein (LDL), uACR or eGFR were excluded. Finally, 1451 participants were eligible and analyzed (Fig. 1). The investigation was conducted according to the Declaration of Helsinki and was approved by the Ethics Committee of Peking University First Hospital. Written informed consent was obtained from all participants.

### Questionnaire assessment

Questionnaire information included demographic and socioeconomic data. Variables including age, gender, smoking status, alcohol consumption, physical activity, education level, history of antihypertensive medicine, lipid-lowering drugs, and antidiabetic medicine, were used in this current study. Smoking status and alcohol consumption were all classified as ‘never’, ‘former/quit’, ‘often’. Physical activity was divided as ‘never’, ‘occasionally’ and ‘often’. Education was classified as ‘illteracy/primary school’, ‘middle school’, and ‘college/university’. Diagnosis and treatment of diabetes, diabetic kidney disease (DKD), and cognitive function were also administrated.

### Anthropometric measurements

Anthropometric indices included height and weight. Height measurement was accurate to 0.1 cm with a tape rule and weight measurement was accurate to 0.1 kilograms with calibrated platform scales. Body mass index (BMI) was calculated as weight (kilograms) divided by height (meters) squared.

### Laboratory assessment

Whole blood samples were drawn from all participants, generally after an overnight fast and analyzed in the Central Laboratory of Kailuan General Hospital on the same day. Plasma samples were used to measure biochemical variables. Triglyceride, TCHO, hemoglobin, HDL, LDL were measured using a Hitachi 7600 auto-analyzer (Hitachi; Tokyo, Japan). Fasting blood glucose (FBG) was tested with the Hexokinase method (BioSino Bio-Technology & Science Inc., China). High sensitivity C reactive protein (hsCRP) was measured using a high-sensitivity particle-enhanced immunonephelometry assay (Cias Latex CRP-H, Kanto Chemical Co. Inc, Japan). Laboratory urine tests including urinary creatinine, urinary albumin, and urinary α1MG, transferrin and α1-acid glycoprotein that regarded as the indices of early DKD [17] were measured in the central laboratory in Peking University First Hospital. Serum creatinine was measured using the Jaffe’s method. eGFR was calculated using the CKD-EPI equation [24].

### Assessment of blood pressure

During the biennial follow-up, BP was measured according to the JNC7 recommendation [25]. After rested in a chair for at least 5 min, BP was measured on the left arm using a mercury sphygmomanometer. SBP is the point at which the first of ≥ 2 Korotkoff sounds is heard, and the disappearance of Korotkoff sound is used to define diastolic blood pressrue (DBP). Two times each of SBP and DBP were obtained at a 5-minute interval and the average value of the two measures was used for further analysis. If the two measurements differed by > 5 mm Hg, then an additional reading was taken, and the average of the three readings was used for data analysis. People with more than three records of SBP recruited in the 2006–2014 examination circles were regarded as the baseline participants in the current study.

### Statistical analysis

Continuous variables were presented as the mean with standard deviation or median with interquartile range. Categorical variables were presented as counts with percentages. Intergroup differences were assessed using one-way ANOVA for normally distributed data. Differences of parameters that were not normally distributed were tested with the Kruskal–Wallis test. Differences of qualitative parameters were compared using the χ^2^ test/Fisher exact test.

The SBP trajectories from 2006 to 2014 biennially were identified by latent mixture modeling (PROC TRAJ) measures which was used to identify subgroups of people sharing similar SBP patterns and the model fit was assessed using the Bayesian information criterion (BIC) [26, 27]. We launched a model with five trajectories and then compared the BIC of the model (-27173.7) to those with 4, 3, 2, and 1 trajectories, respectively. The model with five trajectories was identified with the best fit. We then compared the model in terms of functional forms. Cubic, quadratic, and linear terms were evaluated based on their statistical significance after starting with the highest polynomial. In our final model, we had two trajectories with linear order term and three trajectories with up to quadratic order terms.

Linear regression model, binary logistic regression and multinomial logistic regression model were used to assess the association between SBP trajectory groups and indicators of early kidney damage. For linear regression, uACR, urinary α1MG, transferrin, and α1-acid glycoprotein underwent a logarithmic transformation to make it normally distributed. For logistic regression, urinary albumin to creatinine ratio was divided into three groups (uACR < 3 mg/mmol; 30 mg/mmol ≥ uACR ≥ 3 mg/mmol; uACR ≥ 30 mg/mmol), eGFR was divided into two groups (eGFR ≥ 60 ml/min/1.73 m^2^; eGFR < 60 ml/min/1.73 m^2^) [28], and urinary α1MG, transferrin and α1-acid glycoprotein were all divided according to the value in 75th percentile. The variables included in multivariable regression model included age, gender in model 2; plus history of myocardial infarction (yes vs. no), stroke (yes vs. no), cancer (yes vs. no) in model 3; plus education (illteracy/primary school vs. middle school vs. college/university), physical activity (never vs. occasionally vs. often), smoking status (never vs. former/quit vs. often), drinking status (never vs. former/quit vs. often), triglyceride (a continuous variable), total cholesterol (a continuous variable), BMI (a continuous variable), LDL (a continuous variable), HDL (a continuous variable), hsCRP (a continuous variable) in model 4; plus antihypertensive (yes vs. no), hypoglycemic (yes vs. no), lipid-lowering drug (yes vs. no) in model 5.

We performed the following sensitivity analyses to assess the robustness of our findings. We expanded the recruited population to those with more than 2 times of SBP records during the follow-up period to avoid the selection bias of the population. All *P*-values were calculated based on two-tailed tests of statistical significance. *P* value< 0.05 was considered to be statistically significant. All analyses were conducted using SAS, version 9.4 (SAS Institute, CA, USA).

## Results

**Fig. 2:**
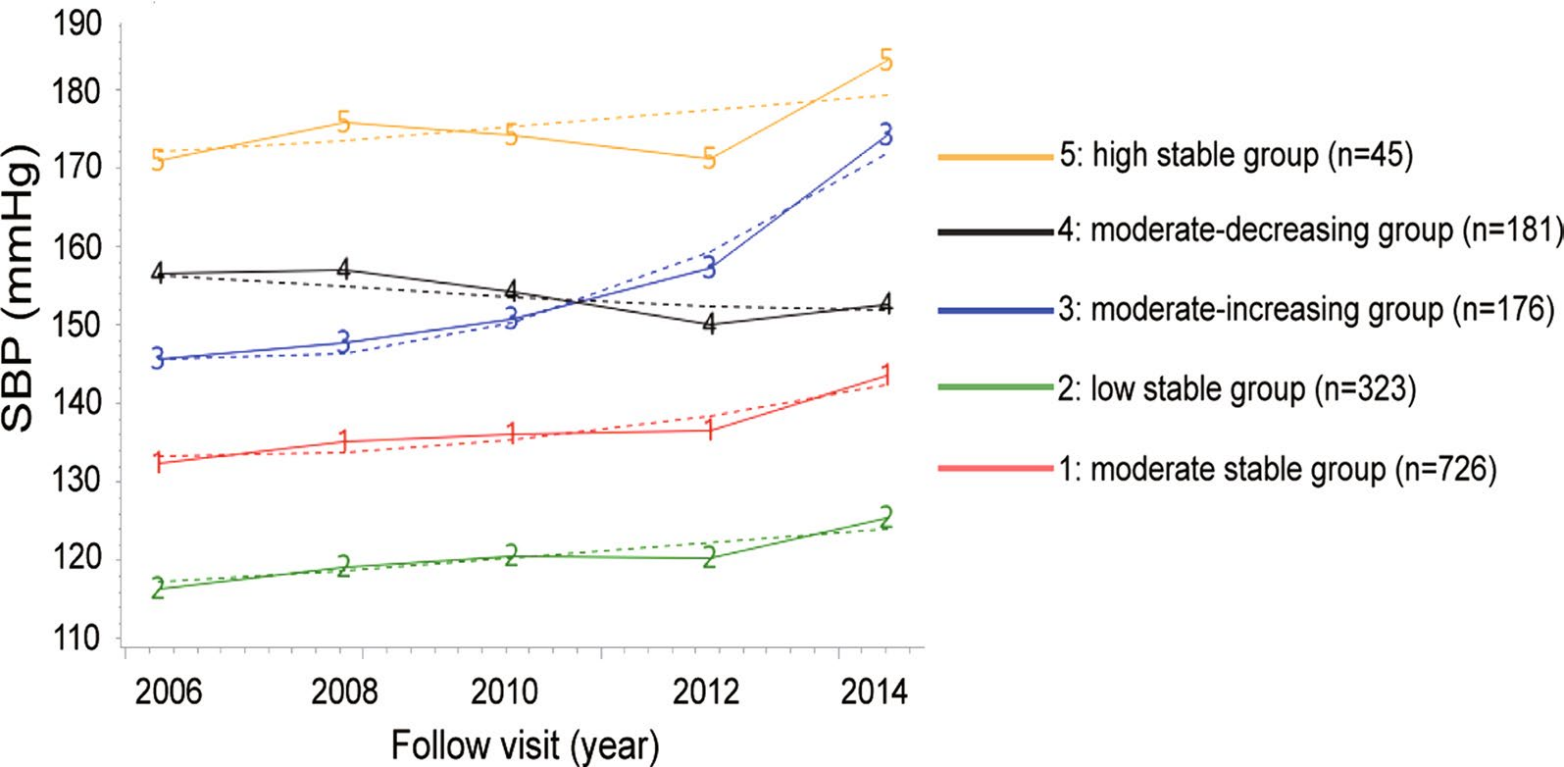
Systolic blood pressure was classified into five groups according to the latent mixture modeling from 2006 to 2014

Altogether, 1451 patients with prediabetes and without kidney damage at the baseline were recruited in this study, with an age of 51.4 ± 8.8 years, and 81.7% (N = 1186) of males. Among the included population in our study, most people remained the status of prediabetes or progressed to diabetes during the follow-up period (259 [17.9%] and 1089 [75.0%], respectively), with only a small proportion having their fasting blood glucose level resumed to the normal range (103 [7.1%]). During the 10-year follow-up, five SBP trajectories were distinctly separated among the 1451 participants with three or more SBP measurements. The patterns of the trajectories were shown in Fig. 2. Altogether, 726 (50.03%) participants were categorized into moderate-stable group with moderate SBP increasing steadily (95% confidence intervals [CI] range from 131.67 mmHg to 133.52 mmHg), 323 (22.26%) into low-stable group (95% CI, 114.19 mmHg-116.56 mmHg), 176 (12.13%) into moderate-increasing group (95% CI 144.24 mmHg-148.57 mmHg), 181 (12.48%) into moderate-decreasing group (95% CI 157.33 mmHg-161.34 mmHg), and 45 (3.10%) into high-stable group (95% CI 165.00 mmHg-176.88 mmHg).

We used individuals in the low-stable group as the reference. People in the other four groups tend to be older, with a higher proportion of males. Compared with the low-stable group, DBP, uACR, and urinary α1MG, transferrin and α1-acid glycoprotein increased and eGFR decreased among the other four groups (all *P* values < 0.01). Meanwhile, compared with the low-stable group, the proportions of never alcohol intake and taking antihypertensive medicine were higher, and the education levels were lower in the other four groups (all *P* values ≤ 0.01). Proportions of use of lipid-lowering drugs were not significantly different among the five groups (Table 1, Additional file 1: Table S3).

**Table 1:** Baseline characteristics of participants in 2006 and outcome indictors in 2016 according to SBP trajectory groups

	Group 1	Group 2	Group 3	Group 4	Group 5	*P* value
Baseline variables: demographic and clinical characteristics						
N (%)	726 (50.03%)	323 (22.26%)	176 (12.13%)	181 (12.48%)	45 (3.10%)	–
Age, years	51.3 ± 8.6	47.3 ± 8.8	55.1 ± 7.1	54.2 ± 8.6	57.0 ± 6.8	< 0.001
Women, %	122 (16.80)	75 (23.22)	28 (15.91)	33 (18.23)	7 (15.56)	0.12
Smoking, (%)						
Never	422 (58.13)	179 (55.42)	105 (59.66)	108 (59.67)	26 (57.78)	0.99
Former/quit	60 (8.26)	32 (9.91)	15 (8.52)	12 (6.63)	4 (8.89)	
Often	244 (33.61)	112 (34.67)	56 (31.82)	61 (33.70)	15 (33.33)	
Alcohol intake, (%)						
Never	391 (53.86)	160 (49.54)	96 (54.55)	102 (56.36)	28 (62.22)	0.01
Former/quit	150 (20.66)	105 (32.50)	38 (21.59)	34 (18.78)	7 (15.56)	
Often	185 (25.48)	58 (17.96)	42 (23.86)	45 (24.86)	10 (22.22)	
Physical exercise, (%)						
Never	50 (6.90)	40 (12.38)	12 (6.82)	16 (8.84)	7 (15.56)	0.003
Occasionally	539 (74.34)	246 (76.16)	127 (72.16)	127 (70.17)	34 (75.56)	
Often	136 (18.76)	37 (11.46)	37 (21.02)	38 (20.99)	4 (8.89)	
Education level, (%)						
Illiteracy/primary	56 (7.71)	14 (4.33)	26 (14.77)	23 (12.70)	5 (11.11)	< 0.001
Middle school	633 (86.59)	239 (73.99)	145 (82.38)	153 (84.53)	39 (86.67)	
College/university	37 (5.1)	29 (8.98)	5 (2.84)	5 (2.76)	1 (2.22)	
Antihypertensive agents, %	69 (9.5)	8 (2.48)	39 (22.16)	68 (37.57)	17 (37.78)	< 0.001
Lipid-lowering drugs, %	60 (8.27)	23 (7.12)	20 (11.37)	22 (12.15)	3 (6.67)	0.42
BMI, kg/m^2^	26.9 ± 3.3	25.9 ± 3.4	27.2 ± 3.1	27.4 ± 3.3	27.4 ± 3.9	< 0.001
Baseline variables: biochemical data						
hsCRP, mg/L	1.0 (0.4, 2.4)	0.9 (0.4, 2.1)	1.1 (0.5, 2.6)	1.4 (0.6, 2.8)	1.0 (0.4, 3.0)	0.008
TCHO, mmol/L	5.1 (4.5, 5.8)	5.1 (4.4, 5.7)	5.2 (4.5, 5.8)	5.4 (4.6, 6.0)	5.0 (4.7, 5.8)	0.088
Triglyceride, mmol/L	1.7 (1.1, 2.5)	1.6 (1.0, 2.4)	1.7 (1.2, 2.5)	1.9 (1.3, 1.8)	1.6 (1.2, 2.5)	0.007
HDL, mmol/L	1.5 ± 0.4	1.5 ± 0.4	1.6 ± 0.4	1.5 ± 0.4	1.6 ± 0.4	0.023
LDL, mmol/L	2.4 (1.8, 2.8)	2.4 (1.9, 2.8)	2.5 (1.9, 3.0)	2.3 (1.8, 3.1)	2.3 (2.0, 2.7)	0.44
FBG, mmol/L	6.2 (5.9, 6.5)	6.1 (5.8, 6.5)	6.1 (5.8, 6.4)	6.1 (5.8, 6.5)	6.1 (5.8, 6.4)	0.051
Hemoglobin, mmol/L	9.4 (8.8, 10.1)	9.3 (8.8, 9.9)	9.4 (8.8, 9.9)	9.4 (8.9, 10.0)	9.4 (8.9, 9.9)	0.21
DBP, mmHg	85.7 ± 9.2	77.3 ± 8.0	91.7 ± 9.3	97.9 ± 11.9	99 ± 11.1	< 0.001
SBP, mmHg	132.6 ± 12.7	115.4 ± 10.8	146.4 ± 14.5	159.3 ± 13.7	170.9 ± 19.8	< 0.001
Outcome variables						
eGFR, ml/min/1.73 m^2^	100.5 ± 12.8	103.3 ± 13.6	94.4 ± 13.4	95.7 ± 14.6	89.4 ± 14.3	< 0.001
uACR, mg/mmol	1.6 (0.8, 3.5)	1.2 (0.7, 2.3)	1.9 (0.9, 5.7)	2.1 (1.1, 5.3)	2.9 (1.4, 8.1)	< 0.001
Orm, mg/L	8.4 (3.6, 20.8)	6.1 (2.8, 15.5)	10.3 (3.9, 27.8)	8.5 (4.1, 24.9)	14.7 (5.6, 44.3)	< 0.001
α1MG, mg/L	16.6 (7.5, 30.0)	13.7 (6.0, 25.7)	16.0 (7.4, 32.8)	16.6 (7.9, 30.8)	26.8 (14.2, 39.8)	0.002
TRF, mg/L	2.2 (2.2, 3.3)	2.2 (2.2, 2.3)	2.2 (2.2, 6.0)	2.2 (2.2, 6.2)	3.7 (2.2, 10.4)	< 0.001

Group 1: moderate-stable group; group 2: low-stable group; group 3: moderate-increasing group; group 4: moderate-decreasing group; group 5: high-stable group

*BMI* body mass index, *eGFR* estimated glomerular filtration rate, *hsCRP* high sensitivity C reactive protein, *FBG* fasting blood glucose, *HDL* high-density lipoprotein, *LDL* low-density lipoprotein, *TCHO* total cholesterol, *DBP* diastolic blood pressure, *SBP* systolic blood pressure, *α1MG* Urinary α1-microglobulin, *TRF* Transferrinuria, *Orm* Urinary α1-acid glycoprotein, *uACR* urinary albumin creatinine ratio

[Missing Value]: Physical activity 1; BMI: 3; Hemoglobin: 3; Diastolic blood pressure (DBP): 1; hsCRP: 1; Orm: 7; α1MG: 5; TRF: 6

Compared with the low-stable group, all the other four groups were associated with a decreased level of eGFR, with the regression coefficients of 0.85, − 1.80, − 1.26, and 4.99, for the moderate-stable, moderate-increasing, moderate-decreasing, and high-stable group, respectively. Higher SBP levels were associated with higher levels of kidney damage markers. For example, the regression coefficients for the logarithm transformed uACR were 0.09, 0.23, 0.25, and 0.35, respectively. The results for logarithm transformed urinary α1-microglobulin, transferrin, and α1-acid glycoprotein were similar (Table 2). In the logistic regression analysis, where eGFR and the four kidney damage markers were under categorization to represent abnormality, the other four SBP trajectories were not significantly associated with eGFR < 60 ml/min/1.73 m^2^, but significant associations were detected between the moderate-decreasing group and uACR categories, between the high-stable group and kidney damage markers of urinary α1-acid glycoprotein > 21.27 mg/L or α1-microglobulin > 29.75 mg/L, between each of the groups (the moderate-increasing group, the moderate-decreasing group or the high-stable group) and transferrin > 3.83 mg/L, respectively, compared with the low-stable group (all *P* values < 0.05) (Table 3).

**Table 2:** Linear regression analysis between SBP trajectory groups and indicators of kidney disease in 2016

	Group 1	Group 2	Group 3	Group 4	Group 5
N (%)	726 (50.3%)	323 (22.3%)	176 (12.1%)	181 (12.5%)	45 (2.8%)
eGFR (n = 1451)					
Model 1	− 2.75^*^	1.00	− 8.90^*^	− 7.62^*^	− 13.85^*^
Model 2	0.96	1.00	− 1.66	− 1.19	− 4.81^*^
Model 3	0.99	1.00	− 1.61	− 1.13	− 4.70^*^
Model 4	0.84	1.00	− 1.86	− 1.34	− 5.06^*^
Model 5	0.85	1.00	− 1.80	− 1.26	− 4.99^*^
Logarithm transformed uACR (n = 1451)					
Model 1	0.09^*^	1.00	0.22^*^	0.27^*^	0.35^*^
Model 2	0.11^*^	1.00	0.23^*^	0.28^*^	0.36^*^
Model 3	0.11^*^	1.00	0.23^*^	0.28^*^	0.36^*^
Model 4	0.09^*^	1.00	0.22^*^	0.24^*^	0.35^*^
Model 5	0.09^*^	1.00	0.23^*^	0.25^*^	0.35^*^
Logarithm transformed Orm (n = 1444)					
Model 1	0.12^*^	1.00	0.16^*^	0.15^*^	0.34^*^
Model 2	0.12^*^	1.00	0.17^*^	0.16^*^	0.36^*^
Model 3	0.12^*^	1.00	0.17^*^	0.16^*^	0.36^*^
Model 4	0.11^*^	1.00	0.16^*^	0.13^*^	0.35^*^
Model 5	0.11^*^	1.00	0.17^*^	0.13^*^	0.35^*^
Logarithm transformed α 1MG (n = 1446)					
Model 1	0.07^*^	1.00	0.07^*^	0.08^*^	0.24^*^
Model 2	0.05	1.00	0.05	0.06	0.22^*^
Model 3	0.05	1.00	0.05	0.06	0.21^*^
Model 4	0.04	1.00	0.05	0.05	0.22^*^
Model 5	0.04	1.00	0.05	0.05	0.22^*^
Logarithm transformed TRF (n = 1445)					
Model 1	0.03	1.00	0.10^*^	0.15^*^	0.20^*^
Model 2	0.03	1.00	0.11^*^	0.16^*^	0.22^*^
Model 3	0.03	1.00	0.11^*^	0.16^*^	0.22^*^
Model 4	0.02	1.00	0.10^*^	0.13^*^	0.21^*^
Model 5	0.02	1.00	0.10^*^	0.14^*^	0.22^*^

Group 1: moderate-stable group; group 2: low-stable group; group 3: moderate-increasing group; group 4: moderate-decreasing group; group 5: high-stable group

Model 1: no adjustment;

Model 2: adjusted for age, gender;

Model 3: further adjusted for history of myocardial infarction, stroke, cancer;

Model 4: further adjusted for education, physics, smoking status, drinking status, salt habit, triglyceride, total cholesterol, body mass index, LDL, HDL, hsCRP;

Model 5: further adjusted for antihypertensive, hypoglycemic, lipid-lowering drug;

*eGFR* estimated glomerular filtration rate, *α1MG* Urinary α1-microglobulin, *TRF* Transferrinuria, *Orm* Urinary α1-acid glycoprotein, *uACR* urinary albumin creatinine ratio

**Table 3:** Logistic regression analysis between SBP trajectory groups and abnormality for indicators of kidney disease in 2016

	Group 1	Group 2	Group 3	Group 4	Group 5
N (%)	726 (50.3%)	323 (22.3%)	176 (12.1%)	181 (12.5%)	45 (2.8%)
eGFR < 60 ml/min/1.73 m^2^					
Model 1	0.66 (0.11, 4.01)	1.00	0.92 (0.08, 10.19)	2.71 (0.45, 16.34)	3.65 (0.32, 41.06)
Model 2	0.41 (0.06, 2.51)	1.00	0.41 (0.04, 4.75)	1.18 (0.18, 7.71)	1.33 (0.11, 16.04)
Model 3	0.41 (0.07, 2.54)	1.00	0.42 (0.04, 4.88)	1.17 (0.18, 7.66)	1.30 (0.11, 15.87)
Model 4	0.32 (0.04, 2.30)	1.00	0.23 (0.02, 3.07)	0.78 (0.10, 5.99)	0.86 (0.06, 11.36)
Model 5	0.32 (0.04, 2.35)	1.00	0.18 (0.01, 2.71)	0.61 (0.07, 5.74)	0.73 (0.05, 10.24)
3 mg/mmol ≤ uACR ≤ 30 mg/mmol					
Model 1	0.99 (0.64, 1.52)	1.00	1.67 (1.02, 1.24)^*^	2.32 (1.52, 3.56)^*^	1.18 (0.41, 3.41)
Model 2	0.97 (0.63, 1.51)	1.00	1.62 (0.97, 2.69)	2.26 (1.45, 3.52)^*^	1.14 (0.39, 3.33)
Model 3	0.97 (0.62, 1.51)	1.00	1.62 (0.98, 2.69)	2.27 (1.46, 3.54)^*^	1.13 (0.39, 3.32)
Model 4	0.90 (0.57, 1.42)	1.00	1.55 (0.90, 2.65)	2.22 (1.38, 3.57)^*^	1.23 (0.41, 3.71)
Model 5	0.90 (0.57, 1.43)	1.00	1.59 (0.92, 2.74)	2.29 (1.40, 3.77)^*^	1.27 (0.42, 3.88)
uACR ≥ 30 mg/mmol					
Model 1	0.79 (0.51, 1.24)	1.00	1.02 (0.61, 1.69)	1.68 (1.06, 2.64)^*^	(0.59 (0.20, 1.71)
Model 2	0.76 (0.48, 1.20)	1.00	0.95 (0.56, 1.60)	1.58 (0.98, 2.52)	0.54 (0.18, 1.59)
Model 3	0.76 (0.48, 1.20)	1.00	0.95 (0.56, 1.61)	1.58 (0.99, 2.54)	0.53 (0.18, 1.58)
Model 4	0.72 (0.45, 1.17)	1.00	0.90 (0.52, 1.58)	1.65 (0.99, 2.73)	0.58 (0.19, 1.75)
Model 5	0.73 (0.45, 1.18)	1.00	0.93 (0.53, 1.64)	1.74 (1.03, 2.93)^*^	0.61 (0.20, 1.86)
Orm > P75 (Orm = 21.27 mg/L)					
Model 1	1.28 (0.93, 2.25)	1.00	1.79 (1.18, 2.73)^*^	1.63 (1.07, 2.49)^*^	3.63 (2.17, 6.08)^*^
Model 2	1.31 (0.94, 1.83)	1.00	1.94 (1.25, 3.01)^*^	1.76 (1.14, 2.73)^*^	3.02 (1.53, 5.95)^*^
Model 3	1.31 (0.94, 1.83)	1.00	1.94 (1.25, 3.02)^*^	1.76 (1.14, 2.73)^*^	3.05 (1.54, 6.01)^*^
Model 4	1.29 (0.92, 1.82)	1.00	1.97 (1.25, 3.10)^*^	1.61 (1.02, 2.54)^*^	3.13 (1.56, 6.27)^*^
Model 5	1.27 (0.90, 1.80)	1.00	1.92 (1.21, 3.05)^*^	1.53 (0.95, 2.46)	2.92 (1.44, 5.94)^*^
α 1MG > P75 (α 1MG = 29.75 mg/L)					
Model 1	1.40 (1.02, 1.93)^*^	1.00	1.64 (1.07, 2.51)^*^	1.45 (0.94, 2.23)	3.02 (1.57, 5.97)^*^
Model 2	1.33 (0.95, 1.85)	1.00	1.56 (0.99, 2.44)	1.40 (0.89, 2.20)	2.95 (1.48, 5.84)^*^
Model 3	1.33 (0.95, 1.85)	1.00	1.56 (0.99, 2.44)	1.39 (0.89, 2.19)	2.92 (1.47, 5.79)^*^
Model 4	1.31 (0.93, 1.85)	1.00	1.52 (0.96, 2.43)	1.31 (0.83, 2.09)	2.96 (1.47, 5.95)^*^
Model 5	1.31 (0.92, 1.85)	1.00	1.53 (0.96, 2.45)	1.31 (0.81, 2.13)	2.91 (1.43, 5.94)^*^
TRF > P75 (TRF = 3.83 mg/L)					
Model 1	1.39 (0.99, 1.95)	1.00	2.28 (1.49, 3.50)^*^	2.61 (1.72, 3.97)^*^	4.56 (2.37, 8.75)^*^
Model 2	1.43 (1.02, 2.02)^*^	1.00	2.46 (1.58, 3.84)^*^	2.80 (1.82, 4.32)^*^	5.04 (2.58, 9.86)^*^
Model 3	1.44 (1.02, 2.02)^*^	1.00	2.47 (1.58, 3.86)^*^	2.80 (1.81, 4.32)^*^	5.09 (2.60, 9.96)^*^
Model 4	1.34 (0.94, 1.91)	1.00	2.48 (1.57, 3.93)^*^	2.50 (1.60, 3.92)^*^	5.28 (2.65, 10.55)^*^
Model 5	1.33 (0.93, 1.89)	1.00	2.46 (1.54, 3.92)^*^	2.42 (1.51, 3.87)^*^	5.08 (2.51, 10.27)^*^

Group 1: moderate-stable group; group 2: low-stable group; group 3: moderate-increasing group; group 4: moderate-decreasing group; group 5: high-stable group

Model 1: no adjustment;

Model 2: adjusted for age, gender;

Model 3: further adjusted for history of myocardial infarction, stroke, cancer;

Model 4: further adjusted for education, physics, smoking status, drinking status, salt habit, triglyceride, total cholesterol, body mass index, LDL, HDL, hsCRP;

Model 5: further adjusted for antihypertensive, hypoglycemic, lipid-lowering drug;

*eGFR* estimated glomerular filtration rate, *α1MG* Urinary α1-microglobulin, *TRF* Transferrinuria, *Orm* Urinary α1-acid glycoprotein, *uACR* urinary albumin creatinine ratio

We did sensitivity analyses by expanding SBP records into more than twice. The results were consistent with the analysis with at least three times of SBP records. (Additional file 2: Figure S1, Additional file 1: Tables S1, and S2).

## Discussion

Five SBP trajectories were identified in this prospective cohort of prediabetes. Overall, higher longitudinal SBP levels based on 10 years of follow-up were associated with subsequent higher levels of markers for kidney damage including uACR, urinary α1-microglobulin, transferrin, and α1-acid glycoprotein, indicating that the current or previous uncontrolled BP was associated with kidney damage. Therefore, long-term dynamic monitoring of BP is necessary for predicting kidney damage in the prediabetic population.

We found that participants in trajectories of moderately or high increased SBP had an adverse spectrum of markers for kidney damage and reduced eGFR. The association was independent of other risk factors for CKD. The results extended the previous findings by providing evidence for the longitudinal pattern of SBP instead of just focusing on the point measure. Derakhshan et al. [9] found that hypertension among prediabetes was associated with abnormal levels for markers of CKD after adjusting for other risk factors for CKD. A recent study in a Chinese cohort showed that the development of hypertension in middle age could increase the risk of CVD [29]. Another study also suggested that higher BP trajectories were correlated with higher uACR [30].

Our results were in accordance with some studies to explain the association between hypertension and kidney damage among patients with prediabetes. Insulin resistance (IR) and decreased beta-cell function have already presented in the stage of prediabetes [31]. IR is associated with hyperfiltration and proteinuria, which links prediabetes and CKD. Otherwise, IR could promote blood pressure contributing to the incidence of CKD [32]. Persistent hyperglycemia promotes proximal tubular reabsorption via type 2 sodium-glucose co-transporter, which induces the decrease of sodium in macula densa with the deactivation of tubuloglomerular feedback and finally increases the glomerular pressure and filtration [12, 13].

DKD is a common complication of diabetes, which manifested as albuminuria and/or decline of eGFR. eGFR indicates the kidney function, and hyperfiltration of glomeruli is responsible for the excretion of urinary albumin. Thus, although albuminuria is considered as a marker for glomerular damage and is an independent risk factor for the progression of CKD, some individuals could only present decreased eGFR and have a normal level of uACR [33, 34]. In the current study, a more comprehensive spectrum of markers were used, including those indicating glomerular injury (transferrinuria), tubular injury (urinary α1MG) and inflammation (urinary α1-acid glycoprotein) [18–20]. These markers can be complementary to the routinely used eGFR and uACR [20, 35, 36]. Part of normoalbuminuric type 2 diabetic patients had transferrinuria and it could be a more sensitive marker of glomerular function [18, 37]. The role of tubular damage and inflammation in kidney disease for patients with prediabetes and diabetes is well known. Studies have shown that urinary α1MG and urinary α1-acid glycoprotein are useful markers of early renal damage among people with abnormally controlled glucose [19, 20]. Our results provided new evidence for the relationship between hypertension and kidney disease by investigating makers of early damage of the kidney.

The current study has some strengths and limitations. Strengths included the large sample size and long-term follow-up. Also, we used longitudinal SBP trajectories and included a spectrum of markers for kidney injury. However, some limitations should be mentioned. First, our study was based on a population with a majority of men and that may limit the generalizability of the study findings to women. Besides, for women, we don’t have records of menopause, so we cannot evaluate the effects of estrogen on blood glucose. Second, patients with isolated impaired glucose tolerance are more than those with isolated abnormal fasting glucose [38], and our study only used the fasting blood glucose to recruit our target population with prediabetes. Thus, some prediabetes people might be excluded by our current criterion. Besides, the records of glycated albumin were lacking, and this parameter may serve as a better indicator for screening prediabetes [39–41]. Third, prediabetes can be induced by lipid-lowering drugs, and change of dose or stop of treatment for such drugs may have introduced bias for the recruited population. However, since the proportion of using lipid-lowering therapy is low and most of patients did not experience remission of prediabetes during the follow-up period, the influence of the bias may be negligible in the current study.

## Conclusions

In summary, patients with persistent high-level SBP or gradually increased SBP could lead to lower level of eGFR and higher levels of markers of kidney damage than those with persistent low SBP. Performing long-term dynamic monitoring for trajectories of blood pressure may be a reliable approach to identify the prediabetic population with a higher risk of DKD.

## Supplementary information

**Additional file 1: Table S1.** Linear regression analysis between SBP trajectory groups and indicators of kidney disease in 2016 among participants with two or more records of SBP. **Table S2.** Logistic regression analysis between SBP trajectory groups and abnormality for indicators of kidney disease in 2016 among participants with two or more records of SBP. **Table S3.** Concomitant therapies between SBP trajectory groups from 2006 to 2012.

**Additional file 2: Figure S1.** Systolic blood pressure was classified into five groups according to the latent mixture modeling from 2006 to 2014 among participants with two or more records of SBP.

## Data Availability

All data generated or analyzed during this study are included in this published article.

## Abbreviations

α1MG: α1-microglobulin
BIC: Bayesian information criterion
BMI: Body mass index
BP: Blood pressure
CI: Confidence interval
CKD: Chronic kidney disease
CVD: Cardiovascular disease
DBP: Diastolic blood pressrue
DKD: Diabetic kidney disease
eGFR: Estimated glomerular filtration rate
FBG: Fasting blood glucose
HDL: High-density lipoprotein
hsCRP: High sensitivity C reactive protein
IFG: Impaired fasting glucose
INDEED: The incidence, development, and prognosis of diabetic kidney disease
IR: Insulin resistance
LDL: Low-density lipoprotein
Orm: Urinary α1-acid glycoprotein
SBP: Systolic blood pressure
TCHO: Total cholesterol
TRF: Transferrinuria
uACR: Urinary albumin creatinine ratio
