# Supplementary material for: Unstably controlled systolic blood pressure trajectories are associated with markers for kidney damage in prediabetic population: results from the INDEED cohort study
Source: J Transl Med. 2020 May 12;18:194. doi: 10.1186/s12967-020-02361-5 (PMC7216344; doi:10.1186/s12967-020-02361-5)
Supplement: Supplementary file 1 — Additional file 1: Table S1. Linear regression analysis between SBP trajectory groups and indicators of kidney disease in 2016 among participants with two or more records of SBP. Table S2. Logistic regression analysis between SBP trajectory groups and abnormality for indicators of kidney disease in 2016 among participants with two or more records of SBP. Table S3. Concomitant therapies between SBP trajectory groups from 2006 to 2012. [file 12967_2020_2361_MOESM1_ESM.docx]

**Table S1. Linear regression analysis between SBP trajectory groups and indicators of kidney disease in 2016 among participants with two or more records of SBP.**

| Sensitive analysis | Group 1 | Group 2 | Group 3 | Group 4 | Group 5 |
| --- | --- | --- | --- | --- | --- |
| N (%) | 753(50) | 336(22) | 187(13) | 175(12) | 45(3) |
| eGFR(n=1451) |  |  |  |  |  |
| Model 1 | -2.54^*^ | 1.00 | -8.36^*^ | -8.04^*^ | -13.12^*^ |
| Model 2 | 0.99 | 1.00 | -1.71 | -1.34 | -4.65^*^ |
| Model 3 | 1.02 | 1.00 | -1.71 | -1.27 | -4.54^*^ |
| Model 4 | 0.91 | 1.00 | -1.86^*^ | -1.40^*^ | -4.85^*^ |
| Model 5 | 0.92 | 1.00 | -1.81^*^ | -1.32^*^ | -4.79^*^ |
| Logarithm transformed uACR(n=1451) | | |  |  |  |
| Model 1 | 0.10^*^ | 1.00 | 0.23^*^ | 0.26^**^ | 0.33^*^ |
| Model 2 | 0.10^*^ | 1.00 | 0.24^*^ | 0.27^**^ | 0.34^*^ |
| Model 3 | 0.10^*^ | 1.00 | 0.24^*^ | 0.27^*^ | 0.34^*^ |
| Model 4 | 0.09^*^ | 1.00 | 0.22^*^ | 0.23^*^ | 0.33^*^ |
| Model 5 | 0.09^*^ | 1.00 | 0.23^*^ | 0.25^*^ | 0.34^*^ |
| Logarithm transformed Orm(n=1444) | | |  |  |  |
| Model 1 | 0.12^*^ | 1.00 | 0.16^*^ | 0.16^*^ | 0.39^*^ |
| Model 2 | 0.11^*^ | 1.00 | 0.16^*^ | 0.17^*^ | 0.35^*^ |
| Model 3 | 0.11^*^ | 1.00 | 0.16^*^ | 0.16^*^ | 0.30^*^ |
| Model 4 | 0.10^*^ | 1.00 | 0.16^*^ | 0.13^*^ | 0.34^*^ |
| Model 5 | 0.10^*^ | 1.00 | 0.16^*^ | 0.14^*^ | 0.35^*^ |
| Logarithm transformed α 1MG(n=1446) | | |  |  |  |
| Model 1 | 0.07^*^ | 1.00 | 0.07 | 0.08^*^ | 0.24^*^ |
| Model 2 | 0.04 | 1.00 | 0.05 | 0.07^*^ | 0.21^*^ |
| Model 3 | 0.04 | 1.00 | 0.05 | 0.07 | 0.21^*^ |
| Model 4 | 0.04 | 1.00 | 0.05 | 0.06 | 0.22^*^ |
| Model 5 | 0.04 | 1.00 | 0.05 | 0.06 | 0.22^*^ |
| Logarithm transformed TRF(n=1445) | | |  |  |  |
| Model 1 | 0.03 | 1.00 | 0.09^*^ | 0.15^*^ | 0.19^*^ |
| Model 2 | 0.03 | 1.00 | 0.11^*^ | 0.16^*^ | 0.19^*^ |
| Model 3 | 0.03 | 1.00 | 0.11^*^ | 0.16^*^ | 0.20^*^ |
| Model 4 | 0.02 | 1.00 | 0.10^*^ | 0.13^*^ | 0.19^*^ |
| Model 5 | 0.02 | 1.00 | 0.10^*^ | 0.14^*^ | 0.20^*^ |

Group 1: moderate-stable group; group 2: low-stable group; group 3: moderate-increasing group; group 4: moderate-decreasing group; group 5: high-stable group.

Model 1: no adjustment;

Model 2: adjusted for age gender;

Model 3: further adjusted for history of myocardial infarction, stroke, cancer;

Model 4: further adjusted for education, physics, smoking status, drinking status, salt habit, triglyceride, total cholesterol, body mass index (BMI), LDL, HDL, hsCRP;

Model 5: further adjusted for antihypertensive, hypoglycemic, lipid-lowering drug;

[Abbreviations] eGFR estimated glomerular filtration rate, α1MG Urinary α1-microglobulin, TRF Transferrinuria, Orm Urinary α1-acid glycoprotein, uACR urinary albumin creatinine ratio.

**Table S2. Logistic regression analysis between SBP trajectory groups and abnormality for indicators of kidney disease in 2016 among participants with two or more records of SBP.**

| Sensitive analysis | Group 1 | Group 2 | Group 3 | Group 4 | Group 5 |
| --- | --- | --- | --- | --- | --- |
| N (%) | 753(50) | 336(22) | 187(13) | 175(12) | 45(3) |
| eGFR<60ml/min/1.73m^2^ | |  |  |  |  |
| Model 1 | 0.67(0.11,4.02) | 1.00 | 0.89(0.08,9.97) | 3.91(0.71,21.54) | 3.79(0.34,42.72) |
| Model 2 | 0.42(0.07,2.58) | 1.00 | 0.44(0.04,5.03) | 1.05(0.28,9.85) | 1.43(0.12,17.25) |
| Model 3 | 0.42(0.07,2.60) | 1.00 | 0.45(0.04,5.16) | 1.64(0.27,9.88) | 1.42(0.12,17.36) |
| Model 4 | 0.32(0.05,2.25) | 1.00 | 0.23(0.02,3.04) | 1.05(0.15,7.23) | 0.89(0.07,11.80) |
| Model 5 | 0.33(0.05,2.34) | 1.00 | 0.24(0.02,3.19) | 1.16(0.16,8.30) | 0.94(0.07,12.68) |
| 3mg/mmol ≤uACR ≤30mg/mmol | | |  |  |  |
| Model 1 | 1.05(0.69,1.60) | 1.00 | 1.65(1.01,2.69)^*^ | 2.40(1.56,3.68)^*^ | 1.18(0.41,3.40) |
| Model 2 | 1.03(0.67,1.58) | 1.00 | 1.60(0.97,2.64) | 2.33(1.49,3.62)^*^ | 1.13(0.39,3.30) |
| Model 3 | 1.03(0.67,1.57) | 1.00 | 1.60(0.96,2.64) | 2.31(1.48,3.61)^*^ | 1.12(0.38,3.26) |
| Model 4 | 0.97(0.62,1.52) | 1.00 | 1.53(0.90,2.61) | 2.29(1.42,3.69)^*^ | 1.22(0.41,3.63) |
| Model 5 | 0.97(0.62,1.53) | 1.00 | 1.56(0.91,2.67) | 2.35(1.42,3.86)^*^ | 1.25(0.41,3.79) |
| uACR≥30mg/mmol | |  |  |  |  |
| Model 1 | 0.86(0.55,1.34) | 1.00 | 1.01(0.61,1.68) | 1.79(1.14,2.82)^*^ | 0.62(0.21,1.81) |
| Model 2 | 0.83(0.53,1.29) | 1.00 | 0.95(0.56,1.59) | 1.68(1.05,2.69)^*^ | 0.57(0.19,1.68) |
| Model 3 | 0.82(0.53,1.29) | 1.00 | 0.94(0.56,1.59) | 1.67(1.04,2.68)^*^ | 0.56(0.19,1.65) |
| Model 4 | 0.80(0.50,1.28) | 1.00 | 0.92(0.53,1.59) | 1.76(1.07,2.92)^*^ | 0.60(0.20,1.81) |
| Model 5 | 0.81(0.51,1.29) | 1.00 | 0.94(0.54,1.64) | 1.85(1.09,3.12)^*^ | 0.63(0.21,1.93) |
| Orm>P75(Orm=21.6mg/L) | |  |  |  |  |
| Model 1 | 1.31(0.96,1.80) | 1.00 | 1.84(1.22,2.77)^*^ | 1.59(1.04,2.43) ^*^ | 2.73(1.42,5.25)^*^ |
| Model 2 | 1.33(0.96,1.83) | 1.00 | 1.96(1.28,3.01)^*^ | 1.73(1.11,2.69) ^*^ | 2.94(1.49,5.78)^*^ |
| Model 3 | 1.33(0.96,1.84) | 1.00 | 1.96(1.28,3.01)^*^ | 1.73(1.12,2.69) ^*^ | 2.97(1.51,5.84)^*^ |
| Model 4 | 1.32(0.94,1.84) | 1.00 | 1.96(1.26,3.06)^*^ | 1.58(0.99,2.50) | 3.04(1.52,6.02)^*^ |
| Model 5 | 1.31(0.94,1.84) | 1.00 | 1.97(1.26,3.07)^*^ | 1.58(0.99,2.52) | 3.04(1.52,6.08)^*^ |
| α 1MG>P75(α 1MG=30.1mg/L) | | |  |  |  |
| Model 1 | 1.36(0.99,1.86) | 1.00 | 1.55(1.02,2.35)^*^ | 0.39(0.90,2.14) | 3.21(1.69,6.13)^*^ |
| Model 2 | 1.27(0.92,1.75) | 1.00 | 1.46(0.94,2.26) | 1.35(0.86,2.12) | 3.01(1.53,5.91)^*^ |
| Model 3 | 1.27(0.91,1.75) | 1.00 | 1.46(0.94,2.26) | 1.34(0.85,2.10) | 2.98(1.52,5.86)^*^ |
| Model 4 | 1.26(0.90,1.75) | 1.00 | 1.42(0.90,2.23) | 1.27(0.80,2.03) | 3.05(1.53,6.06)^*^ |
| Model 5 | 1.25(0.90,1.75) | 1.00 | 1.44(0.91,2.26) | 1.29(0.81,2.07) | 3.08(1.54,6.15)^*^ |
| TRF>P75(TRF=3.8mg/L) | |  |  |  |  |
| Model 1 | 1.38(0.99,1.92) | 1.00 | 2.27(1.50,3.44)^*^ | 2.58(1.69,3.91)^*^ | 4.11(2.15,7.87)^*^ |
| Model 2 | 1.41(1.01,1.96)^*^ | 1.00 | 2.41(1.56,3.69)^*^ | 2.75(1.79,4.25)^*^ | 4.40(2.26,8.57)^*^ |
| Model 3 | 1.41(1.00,1.96)^*^ | 1.00 | 2.41(1.57,3.71)^*^ | 2.74(1.78,4.23)^*^ | 4.42(2.27,8.62)^*^ |
| Model 4 | 1.32(0.93,1.86) | 1.00 | 2.37(1.52,3.70)^*^ | 2.45(1.56,3.83)^*^ | 4.60(2.32,9.13)^*^ |
| Model 5 | 1.32(0.94,1.87) | 1.00 | 2.43(1.56,3.80)^*^ | 2.53(1.61,3.99)^*^ | 4.76(2.39,9.48)^*^ |

Group 1: moderate-stable group; group 2: low-stable group; group 3: moderate-increasing group; group 4: moderate-decreasing group; group 5: high-stable group.

Model 1: no adjustment;

Model 2: adjusted for age gender;

Model 3: further adjusted for history of myocardial infarction, stroke, cancer;

Model 4: further adjusted for education, physics, smoking status, drinking status, salt habit, triglyceride, total cholesterol, body mass index (BMI), LDL, HDL, hsCRP;

Model 5: further adjusted for antihypertensive, hypoglycemic, lipid-lowering drug;

[Abbreviations] eGFR estimated glomerular filtration rate, α1MG Urinary α1-microglobulin, TRF Transferrinuria, Orm Urinary α1-acid glycoprotein, uACR urinary albumin creatinine ratio.

**Table S3. Concomitant therapies between SBP trajectory groups from 2006 to 2012.**

|  | Total* | Group 1 | Group 2 | Group 3 | Group 4 | Group 5 | *P* value |
| --- | --- | --- | --- | --- | --- | --- | --- |
| 2006, (%) | | |  |  |  |  |  |
| Antihypertensive | 201(13.85) | 69(9.5) | 8(2.48) | 39(22.16) | 68(37.57) | 17(37.78) | <0.0001 |
| Lipid-lowering drug | 128(8.82) | 60(8.27) | 23(7.12) | 20(11.37) | 22(12.15) | 3(6.67) | 0.42 |
| 2008, (%) | | |  |  |  |  |  |
| Antihypertensive | 241(20.62) | 84(14.24) | 12(4.30) | 48(36.92) | 76(55.48) | 21(63.64) | <0.0001 |
| 2010, (%) | | |  |  |  |  |  |
| Antihypertensive | 265(22.46) | 118(19.57) | 10(3.64) | 47(34.06) | 80(59.25) | 10(33.33) | <0.0001 |
| Lipid-lowering drug | 17(85.00) | 3(75) | 2(50) | 4(100) | 7(100) | 1(100) | 0.18 |
| 2012, (%) | | |  |  |  |  |  |
| Antihypertensive | 311(25.04) | 140(22.76) | 12(4.29) | 61(39.87) | 84(53.17) | 14(38.89) | <0.0001 |
| Lipid-lowering drug | 14(70.00) | 3(75) | 2(100) | 3(100) | 6(85.71) | 0(0) | 0.04 |

Group 1: moderate-stable group; group 2: low-stable group; group 3: moderate-increasing group; group 4: moderate-decreasing group; group 5: high-stable group.

[Missing Value]: Antihypertensive (2008) 282; Antihypertensive (2010) 271; Lipid-lowering drug (2010) 1431; Antihypertensive (2012) 209; Lipid-lowering drug (2012) 1431;

*: The proportions of the population with concomitant therapies were calculated excluding those with missing values.
