# Supplementary figures and images for: Unstably controlled systolic blood pressure trajectories are associated with markers for kidney damage in prediabetic population: results from the INDEED cohort study
Source: J Transl Med. 2020 May 12;18:194. doi: 10.1186/s12967-020-02361-5 (PMC7216344; doi:10.1186/s12967-020-02361-5)

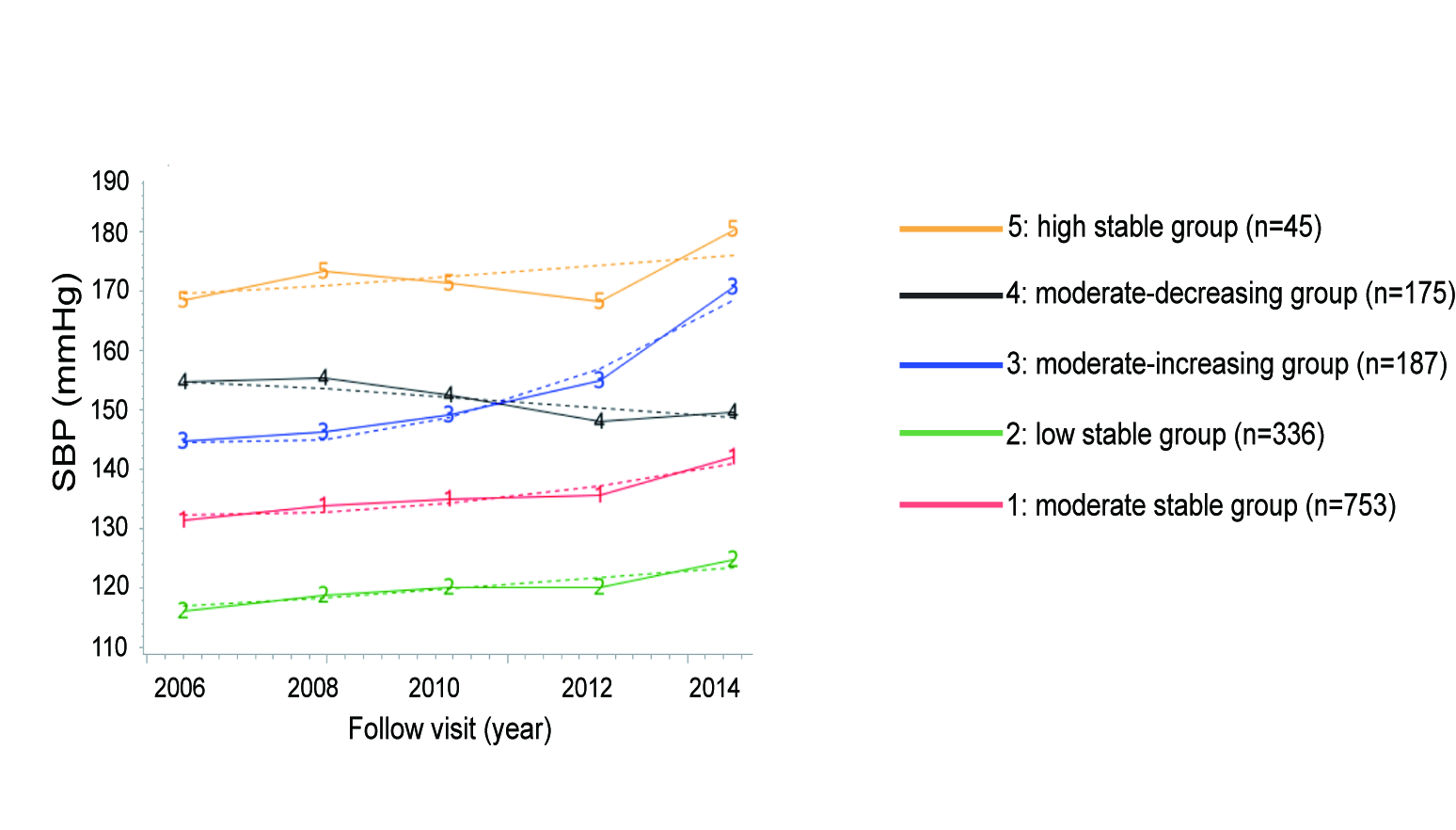

Supplement: Supplementary file 2 — Additional file 2: Figure S1. Systolic blood pressure was classified into five groups according to the latent mixture modeling from 2006 to 2014 among participants with two or more records of SBP. [file 12967_2020_2361_MOESM2_ESM.tif]
